# The Role of Rheological Additives on Fresh and Hardened Properties of Cemented Paste Backfill

**DOI:** 10.3390/ma15093006

**Published:** 2022-04-21

**Authors:** Jiaxu Jin, Zhifa Qin, Shenghao Zuo, Jiaju Feng, Qi Sun

**Affiliations:** 1 School of Civil Engineering, Liaoning Technical University, Fuxin 123000, China; jinjiaxu@lntu.edu.cn (J.J.); 472120681@stu.lntu.edu.cn (J.F.); sunqi@lntu.edu.cn (Q.S.); 2 Liaoning Key Laboratory of Mine Subsidence Disaster Prevention and Control, Liaoning Technical University, Fuxin 123000, China; 3 School of Civil Engineering, Central South University, Changsha 410075, China; zuosh93@csu.edu.cn

**Keywords:** cemented paste backfill, workability, mechanical, drying shrinkage, microstructure

## Abstract

Cemented paste backfill (CPB) has become a significant structural material in most mines across the world. In this study, the effects of chemical rheological additives including viscosity modifying agent (i.e., polyacrylamide) and polycarboxylate superplasticizer (PCE) on fresh and hardened properties of CPB with different water-to-solid (W/S) ratios and water-to-cement (W/C) ratios were investigated. The microstructure of CPB specimens was also characterized by X-ray diffraction (XRD), thermogravimetric analysis (TGA), scanning electron microscopy (SEM), and backscattered electron image (SEM-BSE). The obtained results indicate that PAM (polyacrylamide) dosage and W/S are the most significant parameters influencing the workability of fresh CPB mixtures. For the hardened CPB specimens, the decreasing W/S ratio leads to higher flexural and compressive strength values and lower dry shrinkage strains. The interfacial transition zone (ITZ) between the cement matrix and the tailings sand was also observed to be narrower, with fewer micro cracks and capillary pores. Meanwhile, the existence of PAM decreased the number of hydration products and retarded the hydration reaction. Overall, the CPBs with high W/C ratios (i.e., 1.0 and 1.2), low W/S ratios (i.e., 0.3), and moderate amounts of rheological additives (i.e., 0.05% PAM and 1.0% PCE) have excellent fresh and hardened properties. The findings of this study contribute to better optimization of CPB mixtures in backfill construction, bringing benefits of low costs and low environmental impacts.

## 1. Introduction

Currently, the easiest and most cost-effective method for mine waste disposal is to reuse tailings as cemented paste backfill (CPB) and transfer them to underground cavities [1,2,3]. Generally, the cemented paste backfilling technology tends to be a sustainable way in mining and green mines construction by facilitating the disposal of mine spoils efficiently, which reduces the dangers of tailings dam collapse and promotes the recovery of resources [4,5,6]. The use of CPB in backfilling technology is desirable for mine backfill plants due to its non-classification, non-segregation, and non-sedimentation characteristics [2]. 

Nevertheless, conventional backfilling construction demands large quantities of cement, which is a costly material and also produces large amounts of greenhouse gases such as carbon dioxide, causing serious energy consumption and environmental pollution problems. It is worth noting that the properties of CPB materials can be negatively affected if the cement dosage is excessively low [7,8]. In addition, it is transported through pumping and/or gravity to the mine goaf [9,10]. Mehdizadeh et al. [11] used ultra-fine sediment to replace cement and concluded that ultra-fine sediment could be effectively used at 5–15% replacement levels to manufacture self-compacting mortar. Li et al. [12] used response surface methodology to systematically investigate and illustrate that fly ash, silica fume, sand to binder ratio and water to binder ratio and their partial interactions have remarkable effects on the rheological and mechanical performances of self-compacting mortars, especially the silica fume and sand to binder ratio. In addition, Belibi Tana et al. [13] have shown the yield stress and slump value of uncemented whole tailings backfill paste slurries were positively correlated, and that increasing the solids content would affect their porosity and enhances their strength resistance. Libre et al. [14] investigated the effect of chemical and mineral admixtures on the fluidity, viscosity, and stability of self-compacting mortars at different water/cement ratios, and proposed suitable workability zones for formulating mixtures. Alternatively, the addition of several chemical admixtures to CPB material would not only decrease the dosage of cement, but also enhance its strength and stability performance [2,15].

Therefore, superplasticizers and viscosity modifying agents can be used to prepare the CPB materials, which ensures the workability of the CPB material and thus prevents clogging of the pipes without increasing the backfilling cost [14,16]. Many problems are resolved in the preparation, transportation, and placement steps of CPB using additives. Notably, the performance of CPB is enhanced by reducing segregation and sedimentation. Viscosity modifying agents generally have a negative effect on the fluidity of the CPB, which improves the other performances of the paste mainly through improving the homogeneous distribution of the fine particles and binders in the CPB material [2]. Chen et al. [17] examined the influences of anionic polyacrylamide (PAM) on the properties of CPB and the results indicated that the existence of PAM dramatically enhanced the yield stress and viscosity of fresh CPB mixtures. Bessaies-Bey et al. [18] showed that PAM micro-gels can be adsorbed on cement particles concurrently to establish bridges between them, thereby increasing the macroscopic yield stress. With respect to the increased viscosity and yield stress owing to the viscosity modifying agents, the polycarboxylate superplasticizer (PCE) can be adopted to regulate the workability [19]. Yang et al. [16] indicated that the use of superplasticizers in CPB specimens was beneficial in maintaining/improving the workability and strength properties by decreasing water requirements without lowering the fluidity or raising the amount of cement. Govin et al. [19] investigated the effect of PCE and hydroxypropyl guar (HPG) on cement paste properties and showed that the rheological properties of the cement paste were significantly modified when HPG and PCE were combinedly used. Thus, the use of rheological additives should be optimized through a multi-objective approach. On the other hand, it is possible for even little dosages of additives to have a remarkable effect on CPB. The unreasonable use of additives in the preparation of CPB mixtures not only increases the costs but also has a detrimental effect on the strength and strength efficiency of the CPB [2,20]. Therefore, the use of chemical additives in the preparation of CPBs needs further exploration and investigation. 

Despite the successful application of paste filling technology, the low consistency and low strength of CPB is a challenge for this system. Therefore, the effect of added rheological additives (PAM and PCE) on the performance of CPB was investigated comprehensively and the underlying mechanism was explored. The fresh and hardened properties of CPB were investigated by workability, mechanical property, drying shrinkage, X-ray diffraction analysis (XRD), thermogravimetric analysis (TGA), scanning electron microscopy (SEM), and backscattered electron image (SEM-BSE) tests. The purpose of this study is to investigate the optimum component ratios of rheological additives for rational use in CPB without increasing the cost of backfilling, to achieve a reduction in the use of cement and water, and to improve the workability and hardening properties of CPB.

## 2. Materials and Methods

### 2.1. Materials

**Figure 1:**
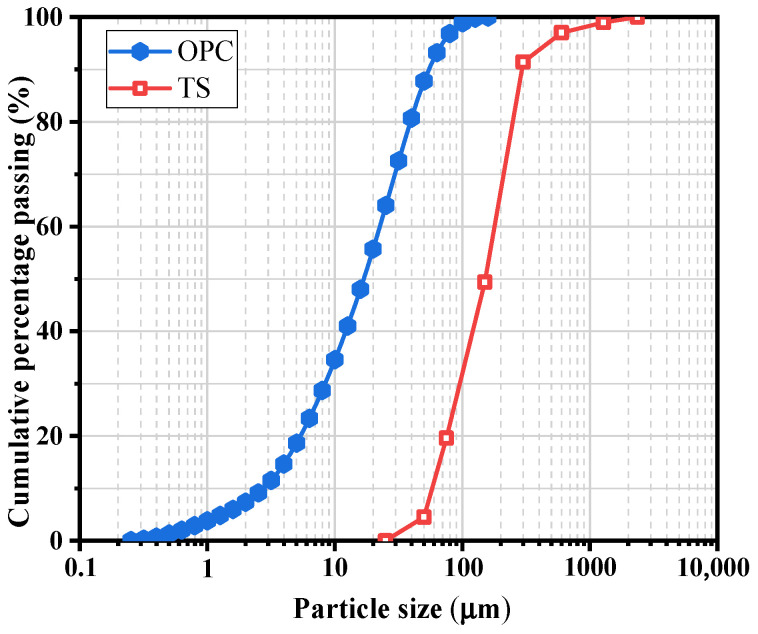
The particle size distributions of OPC and TS.

In this study, P∙O 42.5 ordinary Portland cement (OPC) was used, conforming to GB175-2007. Tailings sand (TS) with a maximum particle size of 2.36 mm and specific gravity of 2.60 from a local iron mine (Chaoyang, Liaoning, China) was used as fine aggregate. The chemical compositions of OPC and TS are summarized in Table 1, respectively, the particle size distributions are shown in Figure 1. The polyacrylamide (PAM) is anionic with a molecular weight of 24 million and was used as the viscosity modifying agent. PCE (polycarboxylate superplasticizer) with a water-reducing rate of 30% was employed to achieve the target workability. All experiments were carried out using local tap water. 

### 2.2. Mixture and Sample Preparations

For the preparation of CPBs, three water-to-solid ratios (W/S, i.e., 0.3, 0.4 and 0.5 by mass) and four water-to-cement ratios (W/C, i.e., 0.6, 0.8, 1.0 and 1.2 by mass) were selected. Initially, the water content of the mixture is ascertained, after which the OPC and TS components were calculated and weighted. The content of PCE was ascertained from the initial flowability of CPBs with different W/S ratios [21]. The detailed mixture proportions are presented in Table 2. The dosages of PAM are 0.05%, 0.10%, and 0.20% by mass of OPC, respectively.

The PAM and PCE (if any) are dissolved in water beforehand. While the OPC and TS are dry mixed in an electric mixer, a solution containing chemical additives is added and mixed at low speed (i.e., 150 rpm) for 1 min and then at high speed (i.e., 300 rpm) for 3 min. Before casting, the fresh CPB mixtures are mixed by hand with a spatula to make them more homogeneous. After the mixing procedure, the fresh CPBs were cast in a 40 × 40 × 160 mm^3^ mold, and the specimens were demolded after 48 h and further cured under standard curing conditions (20 ± 2 °C, 98% RH) until the designed age.

### 2.3. Test Methods

#### 2.3.1. Workability Properties Tests

**Figure 2:**
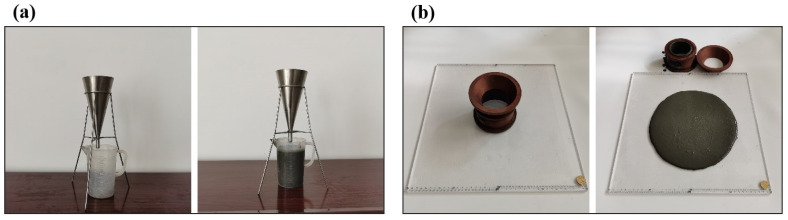
Schematic and photographs of (**a**) Flow time test and (**b**) Flow diameter test.

The measurements of workability properties including flow diameter and flow time were performed on fresh CPB mixtures. The flow diameter of fresh CPBs was determined by the mini-slump method referring to the Chinese standard GB/T 2419-2005. The flow time test was measured with a Marsh Flow Time Meter according to Chinese standard Q/J/11/1999-04. The diameter of the outlet nozzle is 7 mm and the volume of fresh CPB used is 300 mL. The workability test methods of CPBs are shown in Figure 2. More detailed descriptions of test methods and apparatus can be found in our previously published work [21] According to the theoretical and flow velocity of CPBs, the acceptable flow diameter is greater than 25 cm and the flow time is less than 20 s in this study. Moreover, the assessment of CPBs stability in this study complies with the Visual Stability Index (VSI) of ASTM C-1611 [12]. It should be noted here that most of the CPBs mixtures show very slight segregation and bleeding and maintain good stability based on the VSI values.

#### 2.3.2. Mechanical Property Tests

The flexural strength and compressive strength of CPB specimens (40 × 40 × 160 mm^3^) after 28 d of curing were measured according to the Chinese standard procedures given in GB/T 17671-1999. The loading rates for flexural and compressive strength tests are 50 N/s and 2.4 kN/s, respectively. The flexural strength was measured as the average of three measurements and the compressive strength was calculated as the average of six measurements.

#### 2.3.3. Drying Shrinkage Test

The drying shrinkage tests of the CPB specimens were performed in accordance with the JC/T 603-2004. This test determined the change in the length on the drying of the hardened specimens with the dimensions 25 × 25 × 280 mm^3^. The specimens are demolded at 2 d, after further water curing for 1 d, then the specimens were measured daily until 28 days. The drying shrinkage is calculated based on the following Equation (1).

(1) $$L_{i}=\frac{L_{0}-L_{t}}{250}\times 100\%,$$

where *L_i_* is the drying shrinkage of hardened CPB specimen, %; *L*_0_ is the initial length, mm; *L_t_* is the actual length of the test age, mm; 250 is the effective length of the measured specimen, mm.

The percentage mass loss resulting from the evaporation of water was also measured during the drying shrinkage process. The mass loss rate is calculated based on the following Equation (2).

(2) $$S_{28}=\frac{M_{0}-M_{28}}{M_{0}}\times 100\%,$$

where *S*_28_ is the drying water loss rate of hardened CPB specimen at 28 d, %; *M*_0_ and *M*_28_ are the initial mass and actual mass at 28 d, g.

#### 2.3.4. Microstructural Analyses 

The microscopic features of the CPB specimens were identified on the scanning electron microscope (SEM, JSM-7900F, JEOL, Tokyo, Japan) and the Backscattered Electron Image (SEM-BSE, TESCAN MIRA LMS, Brno, Czech Republic). The paste specimens were prepared for XRD tests referring to the mixture proportions of CPBs without using TS. The paste specimens were crushed and ground into powder sample for X-ray diffraction analysis (XRD, Rigaku SmartLab SE, Tokyo, Japan, Cu-Kα), and the test was scanned from 5° to 65° at a rate of 10°/min. The thermogravimetric analysis was conducted on a TGA 2(SF)-Mettler Toledo (Zurich, Switzerland), the powder samples were heated from 35 °C to 1050 °C at a rate of 10 °C/min under the N_2_ flow.

## 3. Results and Discussions

### 3.1. Workability of Fresh CPBs

**Figure 3:**
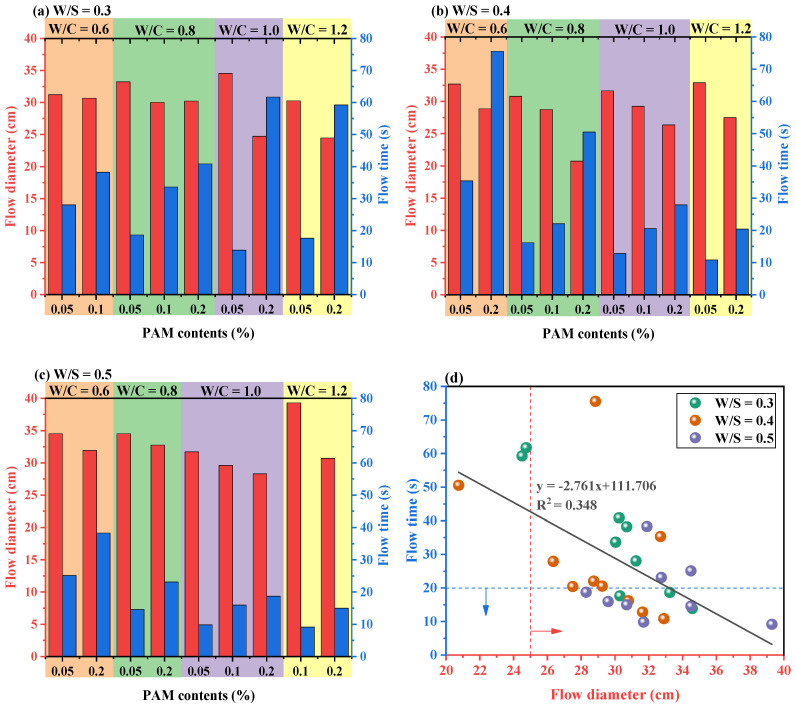
Flow diameter and flow time of fresh CPBs in (**a**–**c**); the fitting relationship of flow time vs. flow diameter in (**d**).

The workability properties are crucial for the transportation and pumping ability of fresh CPBs. In this study, the workability of CPBs were investigated based on flow diameter and flow time tests, and the results are presented in Figure 3. As shown in the figure, the W/C of CPBs is much higher than the conventional cementitious materials, therefore, PAM and PCE were utilized to regulate their workability. The yield stress and viscosity of CPB mixtures significantly improved with the anionic PAM ratio [17]. Yang et al. [16] reported that the used superplasticizers increase the electrostatic repulsion and lubricity between cement particles and improve the fluidity of fresh cemented tailings backfills. However, the excessive amount of PAM and PCE dramatically reduces the workability and hydration rate, which causes negative impacts on casting and mechanical property development [21]. Considering the influences of PAM and PCE in fresh and hardened CPBs, the content of PCE in CPBs is fixed at 1.0%, 0.5%, and 0 for different W/S, respectively, and the content of PAM ranges are 0.05%, 0.10%, and 0.20% to regulate the workability of fresh CPBs.

As seen, the W/S and PAM content exert a significant effect on the workability of mixtures. With an increase in the PAM dosage and solids content, the fluidity of the fresh CPBs decreased significantly, which corresponds to the increasing yield stress and viscosity. Meanwhile, Libre et al. [14] obtained results that also show that using the viscosity modifying agent exerts a deteriorating influence on fluidity. Furthermore, the results showed that using 0.05% to 2.0% PAM decreases the workability of the mixtures linearly (Figure 3). Meanwhile, Ouattara et al. [22] also indicated a well linear relationship between the slump and yield stress of CPB slurries. The flow time is related to the viscosity of fresh CPB and increases with higher viscosity [23]. For fresh CPB mixtures with a W/S of 0.3 (Figure 3a), the addition of PAM reduced the flow diameter and increased the flow time. Consequently, the increased PAM addiction causes a significant increase in the bonding and friction forces between the particles, resulting in higher yield stress and viscosity [17]. Moreover, CPB mixtures with higher W/C displayed lower yield stress and viscosity within a certain range, irrespective of the PAM content, which is attributed to the lubricating function of water [4].

It is well acknowledged that the flow diameter and the flow time tests are available instruments for evaluating the resistance to static segregation of CPB mixtures [12]. To further investigate the workability behavior of fresh CPB mixtures incorporating PAM, the respective flow time results were charted against the associated flow diameter values, as shown in Figure 3d. The flow time versus flow diameter curve is poorly linear with a correlation coefficient of approximately 0.348. The results show that there is no significant linear relationship between the flow time and the flow diameter as the dosage of PAM changes. This poorer linear correlation can be explained by the significant effect in fluidity resulting from the change in W/S, thus increasing the scatter in the data. As can be seen in Figure 3c, the low increment of workability for fresh CPB samples at 0.5 W/S with increased PAM content is partial since its workability is already high without PCE addition [16]. It can be found that the higher solids content (i.e., W/S of 0.3) significantly reduces the workability of CPB. In addition, Qiu et al. [23] observed an amount of binder produced without a significant effect on the fluidity of fresh CPB at the higher solid contents. On the other hand, it can also be seen in Figure 3d that more than 89% of the fluidity of CPB mixtures with different W/S complied with the filling requirements (i.e., flow diameter greater than 25 cm), indicating that the addition of different contents of PCE for the CPB mixtures significantly improved the fluidity. Similarly, Libre et al. [14] showed that the addition of 1% superplasticizer (by weight of the binder) increased the fluidity of the mortar by more than 30%. Therefore, it is possible to add a suitable dosage of PCE to CPB to improve workability or transportability in engineering, owing to the increase in electrostatic repulsion and lubricity between the CPB particles from PCE molecules [4,16].

Based on the above test results and considering the feasibility and cost-effectiveness of backfilling in mine goaf, the final dosage of PAM to be added to CPB specimens with W/S ranging from 0.3 to 0.5 was determined to be 0.05%, 0.05%, and 0.1%, respectively. The rest of the mechanical, drying shrinkage and microscopic tests were conducted using these dosages of PAM. It is worth noting that due to the high solids content of fresh CPB, which often leads to clogging of pipes, controlling the solids content is a widely used preventive measure in engineering practice to ensure the pumpability of fresh CPB [16]. In a further study, the hardening properties of CPB specimens need to be systematically evaluated.

### 3.2. Mechanical Properties of Hardened CPBs

**Figure 4:**
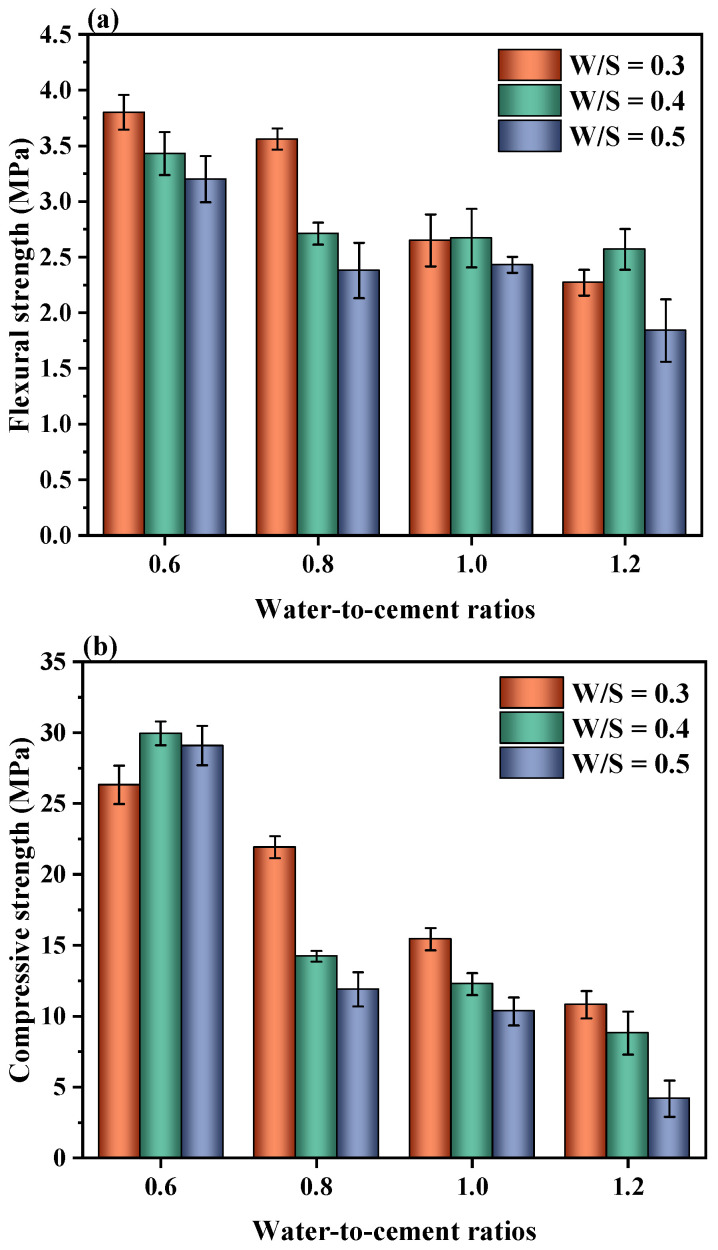
Flexural strength (**a**) and compressive strength (**b**) of hardened CPBs at 28 d.

It is well known that in practical engineering, a balance should be found between the workability and strength of CPB mixture. On the other hand, the hardened CPB must also meet certain mechanical stability expectations to provide safe underground working conditions for all mining personnel. This is because mine backfill failures can not only have considerable economic consequences but can also lead to injuries and/or deaths [9,24]. Therefore, the flexural and compressive strength of CPBs at 28 d was investigated in this section, and the results are shown in Figure 4.

As shown in Figure 4, the strength of CPB is inversely proportional to the W/S of mixtures. The flexural and compressive strength values increase as the solid contents increase. This can be attributed to the cement content per unit volume and total porosity within CPB [16]. The relatively high solid contents clearly increase the strength of CPBs and correspond to lower water contents and the decreased fluidity of fresh CPBs (Figure 3a). In all cases, the mechanical properties of CPB are positively correlated to the W/C ratios, which can be attributed to the higher capillary pore volume originating from the free water in the mixtures. With the increasing W/S, the cement contents per unit volume increase. However, only compressive strength values at W/C of 0.6 show an increasing trend with the cement contents at different W/S ratios, which indicates that total porosity is the main factor influencing the mechanical properties rather than the cement and hydration product content in these cases. Moreover, our previously published work [21], indicated that the addition of PAM has a retarding effect on the hydration of the cement, which can lead to a reduction in strength within certain limits. Meanwhile, Chen et al. [17] also identified that PAM inhibited the strength development of specimens cured for 28 days. On the other hand, OPC content has a significant effect on the mechanical behavior of CPB specimens. As expected, increasing the cement content results in a higher CPB compressive strength (Figure 4b). The highest strength was obtained using the W/C of 0.6 proportion, which is two to three times higher than the strength obtained using the W/C of 1.2 proportion. Güneyisi et al. [25] demonstrated that reducing the water-to-binder ratio significantly increased the compressive strength. Notwithstanding these potential benefits, increasing the amount of OPC will increase the operating costs of CPB, thus in order to reduce the cement content, this study was carried out using rheological additives to modulate the workability and hardening properties of CPB [9].

### 3.3. Drying Shrinkage

**Figure 5:**
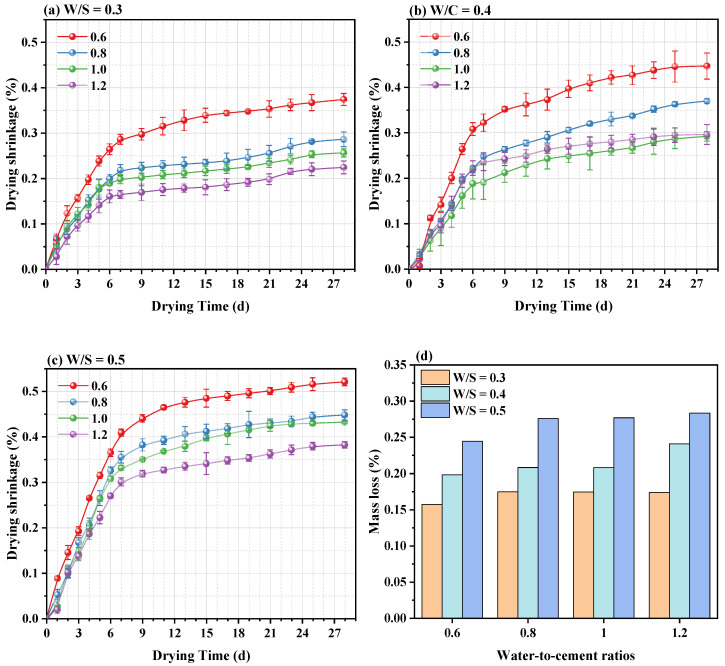
Drying shrinkage (**a**–**c**) and mass loss (**d**) of hardened CPB specimens.

Drying shrinkage is a significant indicator to evaluate the durability performance of hardened CPB, which can be important for the safe operation of a mine after backfilling [26,27]. Furthermore, the drying shrinkage depends on the porosity and elastic modulus [28]. As shown in Figure 5, the drying shrinkage of each CPB sample showed a similar changing tendency, with the shrinkage value increasing faster in the early stage and stabilized in the later stage. The mass loss of specimens with different W/S at 28 d was compared (Figure 5d). It is observed that the mass loss of the specimen decreases with increasing solids content. As expected, the higher the weight loss, the larger the drying shrinkage [28]. Li et al. [26] showed that the water evaporation on the surface of the samples in the early stages mostly from the large capillary pores was caused by the diminution in sample weight. In addition, the existence of macropores causes the relative humidity inside the specimen to drop dramatically without generating sufficiently large capillary stresses. However, the drying shrinkage strain of the specimens was compared in Figure 5a–c. It can be found that the drying shrinkage strain increases significantly with increasing W/S or W/C. Zhang et al. [29] concluded that the drying shrinkage decreases with increasing sand admixture and increases with increasing moisture content. Moreover, the higher the strength grade of the specimen, the lower the drying shrinkage.

### 3.4. Microstructure

**Figure 6:**
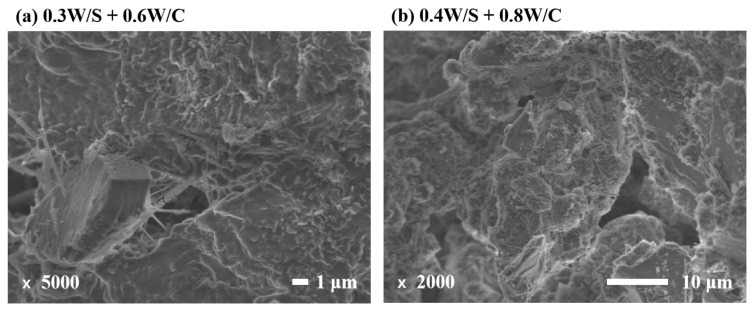

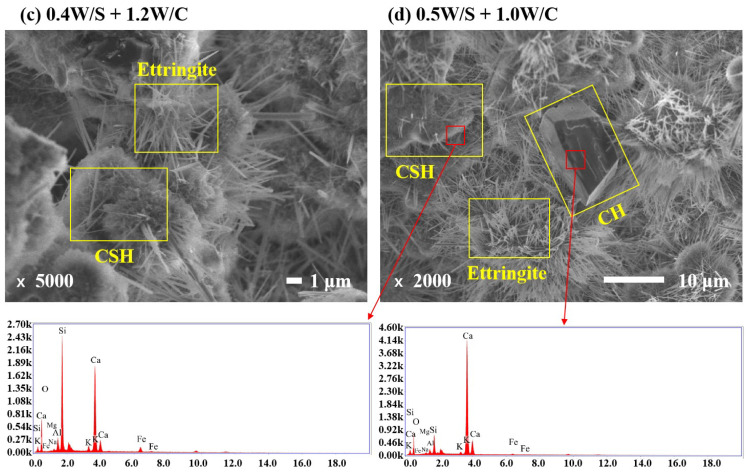
Microstructural features of CPB specimens at 28 d.

Microstructural features allow the study of the microscopic degree of hydration, hydration morphology, and structural details of CPB specimens, which further elaborate the development of macroscopic properties [17]. Figure 6 shows the microstructure of CPB specimens at 28 days under SEM, which presents detailed internal structures of CPB specimens to investigate the calcium silicate hydrate (C–S–H), ettringite, calcium hydroxide (CH), voids, or micro cracks. Indeed, the high molecular polymer PAM molecules connected and interacted with TS and OPC particles by the formation of floc and the bridging effect in CPB mixtures [17]. These floc formed between particles not only enhance the viscosity of fresh CPB mixtures but also increase the mechanical strength of hardened CPB specimens [13]. As shown in Figure 6, the morphologies of hydrates including C-S-H, ettringite, and CH are more relevant to W/S ratios rather than the chemical additives. At high W/S and W/C ratios, there is more available water for cement hydration, and the hydration products are well crystallized, as presented in Figure 6c,d. Moreover, a general trend was observed: TS particles are surrounded by dense hydration products despite a few voids (Figure 6a,b). The water retained in those polymer molecules may promote further hydration to form dense C-S-H, which fill miniature pores and voids and decrease the drying shrinkage of CPB samples (Figure 5) [30]. From another macroscopic perspective, this mechanism is also reflected in the flexural and compressive strength of the corresponding CPB specimen. 

**Figure 7:**
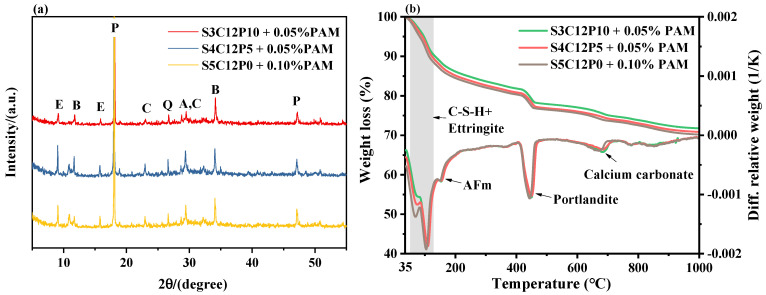
XRD patterns (**a**) and TG−DTG curves (**b**) of hydrated CPB specimens at 28 d (E—ettringite; P—portlandite; C—calcite; Q—quartz; A—alite; B—belite).

To further illustrate the hydration process in CPB containing chemical admixtures, the solid phase composition of the hydrated paste was examined. Figure 7a shows the XRD pattern at 28 days of hydration, where the main crystalline phases from hydration products and raw materials found were ettringite (E), portlandite (P), calcite (C), and quartz (Q), in addition to alite (A) and belite (B) peaks for the unhydrated cement clinkers. Therefore, the use of chemical additives does not change the main hydration products in blends but may affect the hydration process and degree. As shown in Figure 7b, the decomposition peaks at different temperatures correspond to different hydration products. With the increasing W/S ratios, the measured portlandite contents are very close, however, the chemically bound water (i.e., mass loss between 35 °C and 550 °C) contents show an increasing trend, which indicates that a higher content of available water promotes the cement hydration, however, the hydration of silicate clinkers may be retarded owing to the higher PAM contents, but the hydration of aluminate clinkers is not affected, thereby leading to higher chemically bound water content. According to the mechanical results, the improved hydration degree actually shows no obvious benefit, as the porosity of CPB specimens is the dominant parameter.

**Figure 8:**
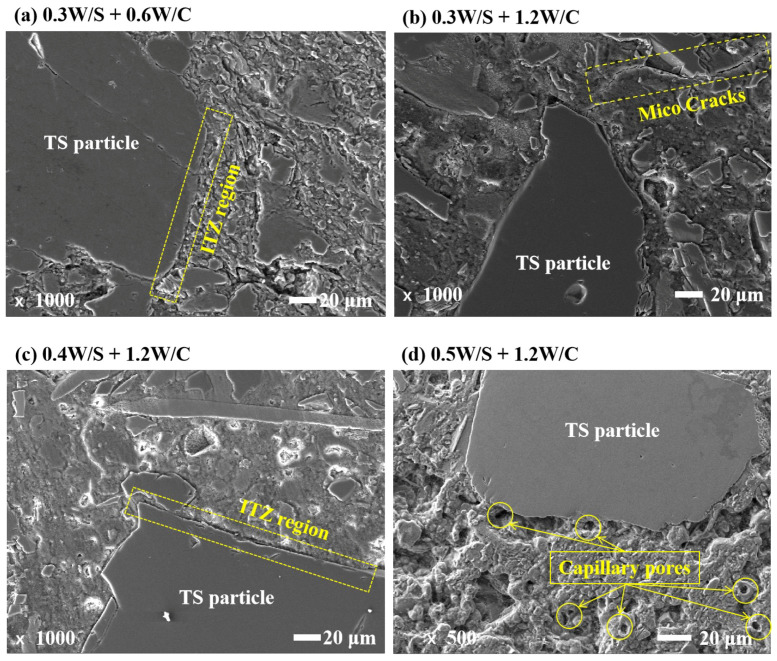
Representative SEM-BSE image of CPB specimens at 28 d.

The interfacial transition zone (ITZ) is a relatively complex and heterogeneous region that is important at the macroscopic scale for the overall assessment of material properties [31]. In hardened CPB materials, the spaces between the TS are filled with hardened hydration products consisting of an ITZ layer surrounding each TS grain and a bulk slurry located outside the ITZ region and further from the TS surface [32,33]. A representative area of CPB at low magnification is shown in Figure 8 and the TS grains are visible. The rheological additives improve CPB performance by improving the interaction between the fine particles in the structure of the mixture. It improves the uniformity of paste and remarkably reduces the “layer structure” inside the paste [2], with a significant reduction in the number of micro-cracks and pores. Chen et al. [17] also concluded that an increase in the proportion of PAM additives resulted in larger floccule links and a more stable paste structure.

Nevertheless, it is possible that a dense ITZ may coexist with a porous ITZ, given that CPB is a highly heterogeneous material with different distances between adjacent TS particles and different properties of the ITZ surrounding the TS particles (Figure 8d) [31]. The permeable micro-cracks exist in Figure 8b, which is an important element in the degradation of the mechanical and durability properties of the specimen. Moreover, compared to CPB specimens with a W/S of 0.3 and a W/S of 0.5, the former has fewer capillary pores and a denser microstructure than the latter, as shown in Figure 8b,d. These results may explain why the CPB specimens with high W/S ratios have lower compressive strengths and higher drying shrinkage strains.

In summary, using a viscosity modifying agent is a very effective tool for stabilizing CPB, and preventing bleeding and segregation. Moreover, the low W/S may be more suitable for CPB application because the yield stress is low enough to allow flow and the paste is more viscous to improve stability. In practice, the selection of CPB mixtures with low W/S using the appropriate amount of chemical additives improves the fresh and hardened properties of CPB, while contributing to the reduction of costs and environmental impact.

## 4. Conclusions

The influence of rheological additives on backfilling the fresh and hardened properties was investigated by various methods. Based on the results and discussion above, the following conclusions can be drawn:

(1) The workability of fresh CPB mixtures mainly depends on the PAM dosage and W/S of mixtures. The yield stress and viscosity of fresh CPB mixtures increased with the PAM dosages. Moreover, reducing the W/S can effectively improve the stability of CPB mixtures;
(2) The strength development of CPB at low W/S is improved, and the consistency of fresh CPB mixtures is positively correlated with the free water content. Reducing the W/S ratio produces a positive effect on the mechanical properties of hardened CPB mixtures, but normally exerts a negative influence on the workability of fresh CPB mixtures;
(3) The higher drying shrinkage and mass loss of CPBs with a W/S ratio of 0.5 compared to that with a W/S ratio of 0.3 are due to the higher capillary pores volume; the increasing W/C ratios of CPBs lead to lower shrinkage strain but slightly higher mass loss during drying, which is attributed to the higher capillary pores volume and the larger mean pore size;
(4) Microstructural analysis reveals that the PAM declines the hydration rate and hydration products amount. With decreasing W/S of the CPBs, the narrower ITZ layers can be achieved. Micro cracks and capillary porosity are other important factors affecting the mechanical and drying shrinkage properties.

## Figures and Tables

**Table 1:** The chemical compositions of OPC and TS.

Chemicals (wt/%)	CaO	SiO_2_	Al_2_O_3_	Fe_2_O_3_	SO_3_	MgO	Other Oxides	LOI
OPC	62.80	20.60	4.13	2.99	2.56	1.93	3.93	1.06
TS	7.31	47.56	7.12	24.65	-	5.42	7.13	0.81

**Table 2:** Mixture proportions of CPBs.

Sample	Water-to-Solid Ratios	Water-to-Cement Ratios	Water (g)	OPC (g)	TS (g)	PCE (%)
S3C06P10	0.3	0.6	900	1500	1500	1.0
S3C08P10		0.8	900	1125	1875	1.0
S3C10P10		1.0	900	900	2100	1.0
S3C12P10		1.2	900	750	2250	1.0
S4C06P5	0.4	0.6	900	1500	750	0.5
S4C08P5		0.8	900	1125	1125	0.5
S4C10P5		1.0	900	900	1350	0.5
S4C12P5		1.2	900	750	1500	0.5
S5C06P0	0.5	0.6	900	1500	300	0
S5C08P0		0.8	900	1125	675	0
S5C10P0		1.0	900	900	900	0
S5C12P0		1.2	900	750	1050	0

Note: S stands for the W/S ratio; C is the W/C ratio; P stands for the PCE content in the mixture. S5C12P0 means that the W/S ratio is 0.5, the W/C ratio is 1.2, and no PCE is used.

## Data Availability

Date can be obtained from corresponding authors upon reasonable request.
